# Dataset for developing deep learning models to assess crack width and self-healing progress in concrete

**DOI:** 10.1038/s41597-025-04485-z

**Published:** 2025-01-28

**Authors:** Jacek Jakubowski, Kamil Tomczak

**Affiliations:** Department of Civil & Geotechnical Engineering and Geomechanics, AGH University of Krakow, al.Mickiewicza 30, 30-059 Krakow, Poland

**Keywords:** Civil engineering, Composites, Scientific data, Imaging and sensing

## Abstract

The presented dataset comes from an experimental study on the autogenous self-healing of high-strength concrete and the development of deep learning metasensor for crack width assessment and self-healing evaluation. Concrete specimens were prepared, matured, cracked, and exposed to self-healing. High-resolution scanning of the specimen surface and scale-invariant image processing were performed, multiple grid lines crossing cracks were established, and brightness degree profiles along grid lines were extracted. Then, reference measurements of the crack widths were obtained by an operator. The dataset comprises 19,098 records of brightness profiles, reference measurements, and benchmark measurements by deep learning and analytic models. The source images, stacked and marked with grid lines, are provided. The considerable number of brightness profiles coupled with manual reference measurements make the dataset well suited for developing an image-based deep CNN models or analytic algorithms for assessing crack widths in concrete. The technical validation study explored three factors that affect crack measurement: the specimen position in relation to the scanner, the surface moisture level, and the operator performing manual measurements.

## Background & Summary

The detection and estimation of crack width in concrete are important in numerous applications in civil engineering, particularly in monitoring the structural health of bridges^1–6^, tunnels^7–12^ and pavements^13–16^. Self-healing is the process of restoring the impermeability and mechanical strength of concrete after cracking occurs. Studies of the self-healing of concrete are still largely in the laboratory phase rather than in the full-scale implementation phase, although research is progressing rapidly. Evaluating and monitoring the progression of the self-healing process are necessary for these studies. Numerous semiautomated and automated crack width measurement approaches using high-resolution images or scans of cracked surfaces have been developed^17^. Nevertheless, the visual examination of cracks at the surfaces of cementitious materials and manual crack width measurements remain the most common evaluation and monitoring techniques. Crack widths are assessed from images using analytic algorithms based on the brightness threshold^1,18,19^, gradients^2,20,21^, Fourier and wavelet transformations^21,22^ or deep learning models^3,4,9,10,13–16,21,23,24^. A deep convolutional neural network (CNN) automatically extracts features from images, enabling the assessment of crack widths based on a training sample. In visual crack width assessment, several specific features, requirements, and factors affect the measurements.

Tomczak *et al*.^17^ presented a semiautomated crack width measurement method that combines preprocessing with a scale-invariant feature transform (SIFT) and an analytic edge detection algorithm (AED). The images acquired after the subsequent stages of self-healing are covered with a dense network of grid lines transversely crossing the cracks. Along the gridlines, brightness degree profiles are extracted for crack width assessment, and two analytic algorithms based on the first and second derivatives of the profiles are applied. This approach enables the self-sealing process to be monitored at many of the same locations after each subsequent observation stage. This is an important feature for precisely assessing self-healing progress. Recently, valuable datasets for the development of machine learning models to aid in the detection and assessment of crack geometry in various engineering structures have become available^25–29^. Crack features such as width or area, detection accuracy or noise reduction in the images were evaluated^21^. Jakubowski and Tomczak^30^ introduced a deep learning metasensor (DLM) to measure crack width from high-resolution images and brightness profiles. The DLM was trained and evaluated using a dataset of 19,098 brightness profiles and reference crack width measurements, and the proposed deployment procedure included data drift evaluation and fine-tuning. Manual reference measurements were obtained with the images at the exact locations of the extracted brightness profiles. This allowed the coupling of brightness profiles (one-dimensional images) with reference crack width measurements, and the development of a metasensor for semiautomatic, multilocation, and multistage crack width measurements.

**Fig. 1:**
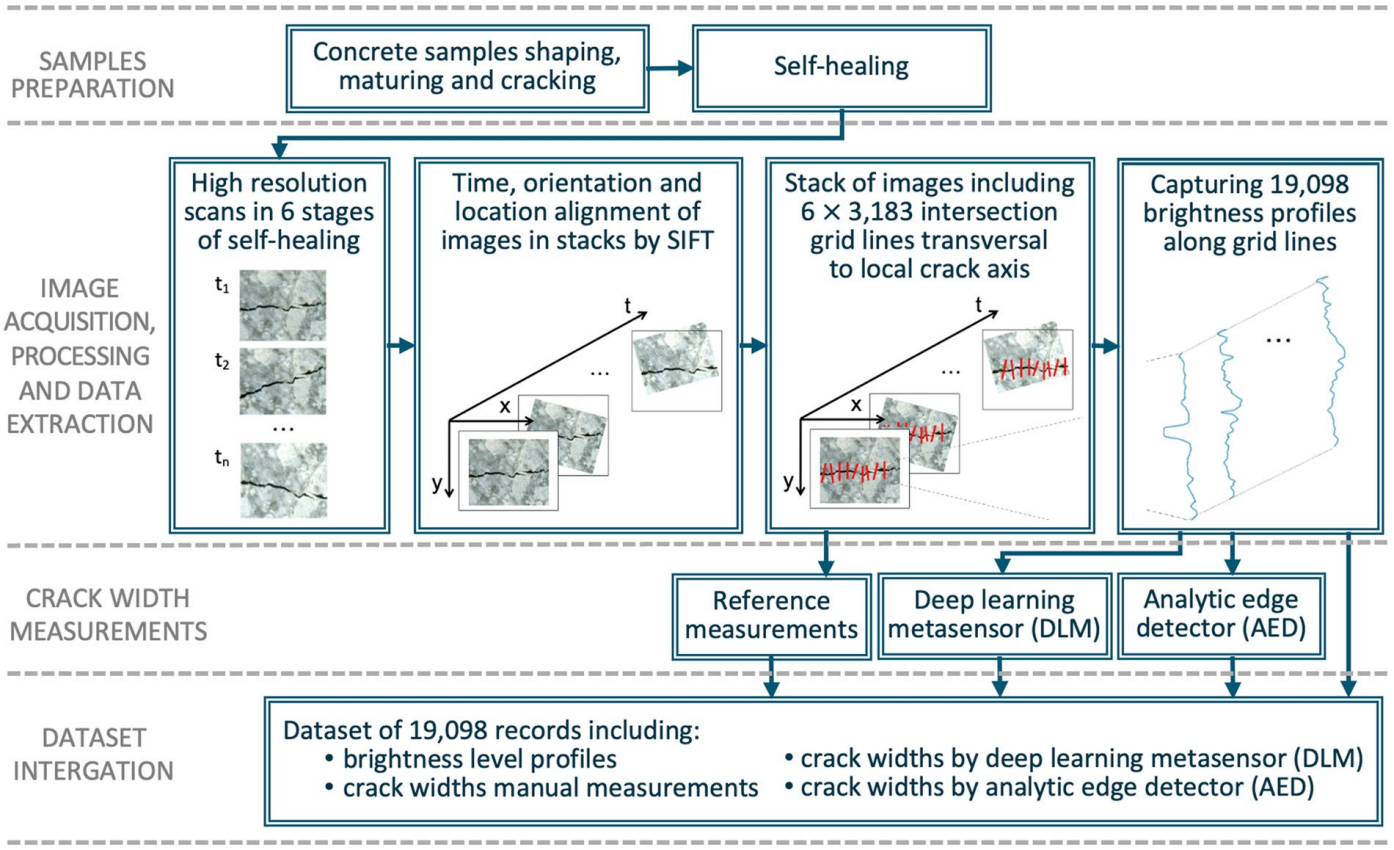
Structure of the study showing the data extraction operations used to produce the provided dataset.

This data descriptor complements the article of Jakubowski and Tomczak^30^; it provides a dataset, source images, and technical details of the data acquisition. High-resolution images are pre-processed, brightness profiles are captured, and reference and benchmark measurements are performed, as shown in Fig. 1. This dataset is unique because of its acquisition methodology, the type of data (crack brightness profiles), and the very large number of manual crack width measurements. The dataset can be used to train or pre-train new deep learning models or analytic algorithms for assessing crack width in concrete, without requiring laboratory experiments and manual measurements. These studies could aid in improving the durability and sustainability of concrete structures by enhancing the monitoring and understanding of self-healing mechanisms. The crack width assessment methodology has a range of possible applications, however, it has been designed for, and is particularly useful for, self-healing investigations. It provides the ability to monitor each subsequent stage of self-healing process in a large number of fixed locations. Thus, it enables accurate estimates and sensitive statistical inference on the self-healing progress.

## Methods

### Specimen components, curing, and cracking

High-strength concrete mixtures (Table 1, CMd_0.28, and CMd_0.23) that are 2 and 20 months old at the time of crack induction are examined. Typically, the cement content of high-strength concrete formulations exceeds that of conventional concrete mixtures by at least a factor of two. A high cement content combined with a very low water content due to the use of an admixture with a high water reduction range leads to the inclusion of nonhydrated cement particles in the hardened concrete. These nonhydrated cement particles function as substrate reservoirs, which are crucial for autogenous self-healing.

**Table 1:** Components of the concrete mixtures and their contents [kg/m^3^ of concrete]^30^.

Component	CMd_0.28	CMd_0.23
CEM I 42.5 R	626.9	819.7
0–2 mm sand	658.3	573.8
Dolomite crushed-stone aggregate 2–8 mm	1117.6	974.5
Water	157.1	159.9
Superplasticiser – CHRYSO® Fluid Optima 185	19.0	24.6
Water-binder ratio	0.28	0.23

The concrete specimens are prepared in ∅ 150 mm × 30  mm cylindrical steel moulds. After 2 days of curing, the cylindrical specimens are demoulded and cured for 7 days at a temperature of 20 ± 2 °C and a relative humidity of 90 ± 5% under a plastic cover. Then, the specimens are placed in a water bath for 2 or 20 months at a temperature of 20 ± 2 °C. After the ageing period, the cylindrical specimens are cut into four slices measuring ∅ 150 mm × 75  mm. The sidewalls of all the specimens are protected by five layers of fiberglass-reinforced tape. Next, crack sizes in the range of 0–800 μm are induced in a splitting test using a Controls Automax 5 testing machine at a loading speed of 125 N/s. The material is prepared in this manner to provide diversity in the composition of the high-strength concrete, the age of the composites at the time of crack induction, and the geometry of the cracks.

### High-resolution scanning of specimen surfaces

Images of all the internal surfaces of the specimen slices are digitally recorded using an Epson Perfection V600 Photo scanner with a CCD matrix, a 6400-dpi optical resolution and an optical density of 3.4 Dmax. The distance between the CCD matrix and the scanned surface is constant, and the pixel size is 3.96875 µm. The specimens are scanned immediately after crack induction (time point t_1_ = 0) and after t = 2, 4, 7, 14, and 28 days (time points t_2_–t_6_) of the self-healing process. Between observations, the specimens are kept in water at 20 ± 2 °C to induce self-healing. Before scanning, the specimens are removed from the water and dried at 24 ± 2 °C for 2 h to reduce surface moisture. To eliminate the influence of varying ambient lighting, each scan is performed under a black matte and an opaque mat. No image correction is performed during scanning. If the surface image does not cover the entire brightness range (0–255), it is rescanned. The surface scans are stored on disks as TIFF files. As a major component of the tests, a total specimen area of 2100 cm^2^ is selected for 16 specimens of CMd_0.28 and CMd_0.23.

### Scale-invariant image preprocessing

Image preprocessing is performed using the ImageJ2 (version 2.9.0/1.53t) programming environment^31^. Images of the same area on the specimen surface are recorded in a chronologically ordered stack. Then, the same coordinate system is applied to all the packed images using the SIFT method^32^. Stacking and processing using SIFT are performed using the recommended parameters listed in Table 2. This is considered important because the self-healing process alters crack appearance at each stage.

**Table 2:** Set of recommended input parameter values for adjusting images using the SIFT method in ImageJ2^30^.

**Scale-invariant interest point detector**	Initial Gaussian blur	1.6 pixels
	Steps per scale octave	3
	Minimum image size	64 pixels
	Maximum image size	1024 pixels
**Feature descriptor**	Feature descriptor size	4
	Feature descriptor orientation bins	8
	Closest/next closest ratio	0.92
**Geometric consensus filter**	Maximal alignment error	25 pixels
	Inlier ratio	0.05
	Expected transformation	Rigid
	Output interpolation	Yes

The SIFT algorithm can be divided into several stages. First, a scale-space pyramid of the obtained image is created. This implies that the original image is repeatedly blurred with the Gaussian filter, generating images of different resolutions. Moreover, this enables the detection of features at different scales, which is a fundamental aspect of the algorithm. The algorithm then detects extremes in scale space. At each scale, the local areas of the image are analysed to identify the feature points. These points are where the gradient of the image reaches its maximum or minimum in a specific area and at a given scale. Second, the orientation of the features is determined. For each feature point, the algorithm calculates the orientation based on the image gradient around the point. This allows the rotation of the object and renders the features invariant to changes in orientation. Finally, the algorithm generates a feature description by dividing the area around the feature point into smaller subregions and calculating the gradient histograms for each of these subregions. The histograms are then combined into a unified vector constituting the feature description. The feature description vectors are normalised to ensure invariance to changes in illumination and contrast. In addition, they are reduced to a fixed length, making it easy to compare the features among different images.

### Crack detection and region of interest determination

**Fig. 2:**
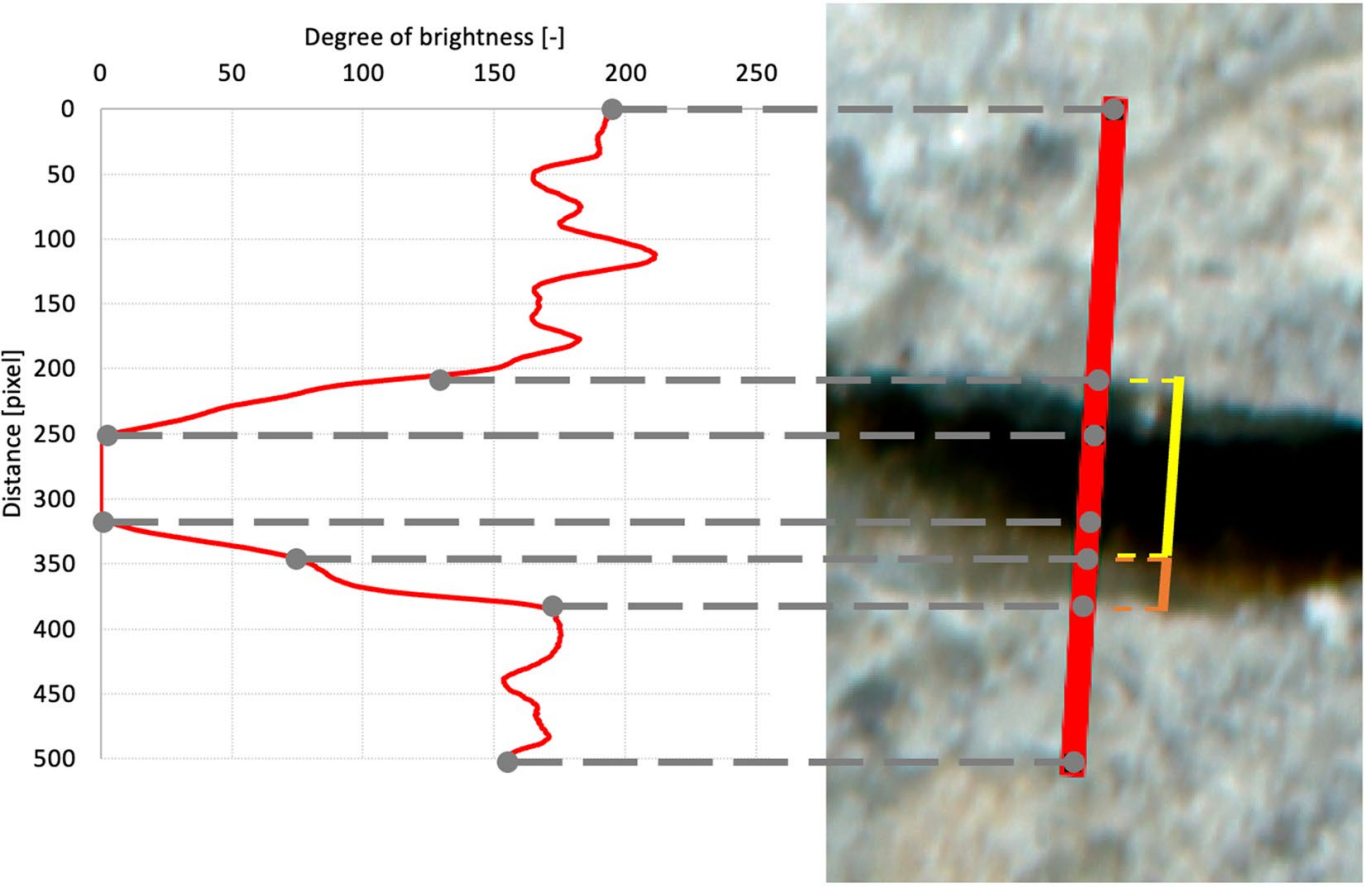
An example of a brightness profile for a crack grid line along with characteristic points visible in an image of the concrete surface. The width of the crack measured by the operator is marked by the yellow line; the orange line indicates the bevelled area of the crack edge.

**Fig. 3:**
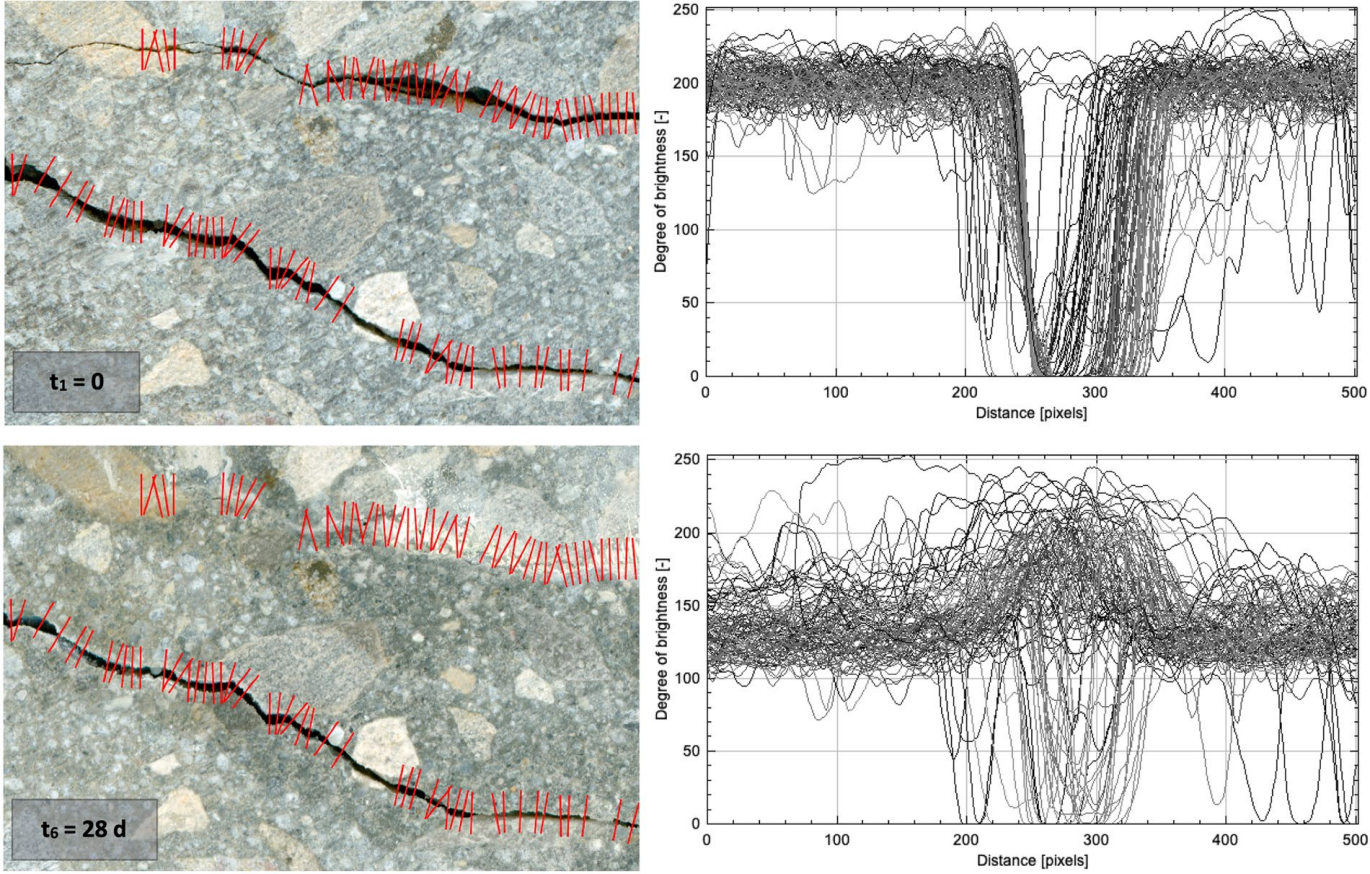
Scans of a specimen with cracks that formed immediately after induction (t_1_ = 0) and after self-healing (t_6_ = 28 d), along with grid lines and degree of brightness profiles. A clear change in the brightness degree profiles can be seen after 28 days of curing, when new material can be observed in the area of cracks.

Using the procedures described by Tomczak *et al*.^17^, the prepared image stacks are input into an algorithm that identifies points with the minimum brightness, corresponding to black in crack areas. The search for points that meet the criteria is performed based on the coordinate system introduced in the image. Crack detection is performed for the first image in the stack, corresponding to the moment immediately after crack induction. The detected points along the cracks are automatically covered by a grid of intersecting lines with a spacing of no less than 100 pixels between them as regions of interest (ROIs). Subsequently, the grid lines are automatically rotated such that they are perpendicular to the local crack axis. The required rotation angle is determined based on the coordinates of the next-closest detected point in the crack area. Finally, the angle of rotation of each grid line is determined based on the position of the nearest preceding point. Subsequently, the brightness profiles of the cracks along all the grid lines at all self-healing stages are extracted. The results are compiled in a dataset of brightness profiles (one-dimensional images) of the crack cross-sections Figure 2 shows an example of a brightness degree profile with characteristic points. Figure 3 contrasts a specimen and brightness profiles immediately after crack induction and after 28 days of self-healing.

### Reference measurements of crack widths

The manual measurement of crack width is based on the visual inspection of crack grid lines on preprocessed images. For this study, 19,098 manual measurements are performed using the ImageJ2 programming environment and graphical user interface^31^.

**Fig. 4:**
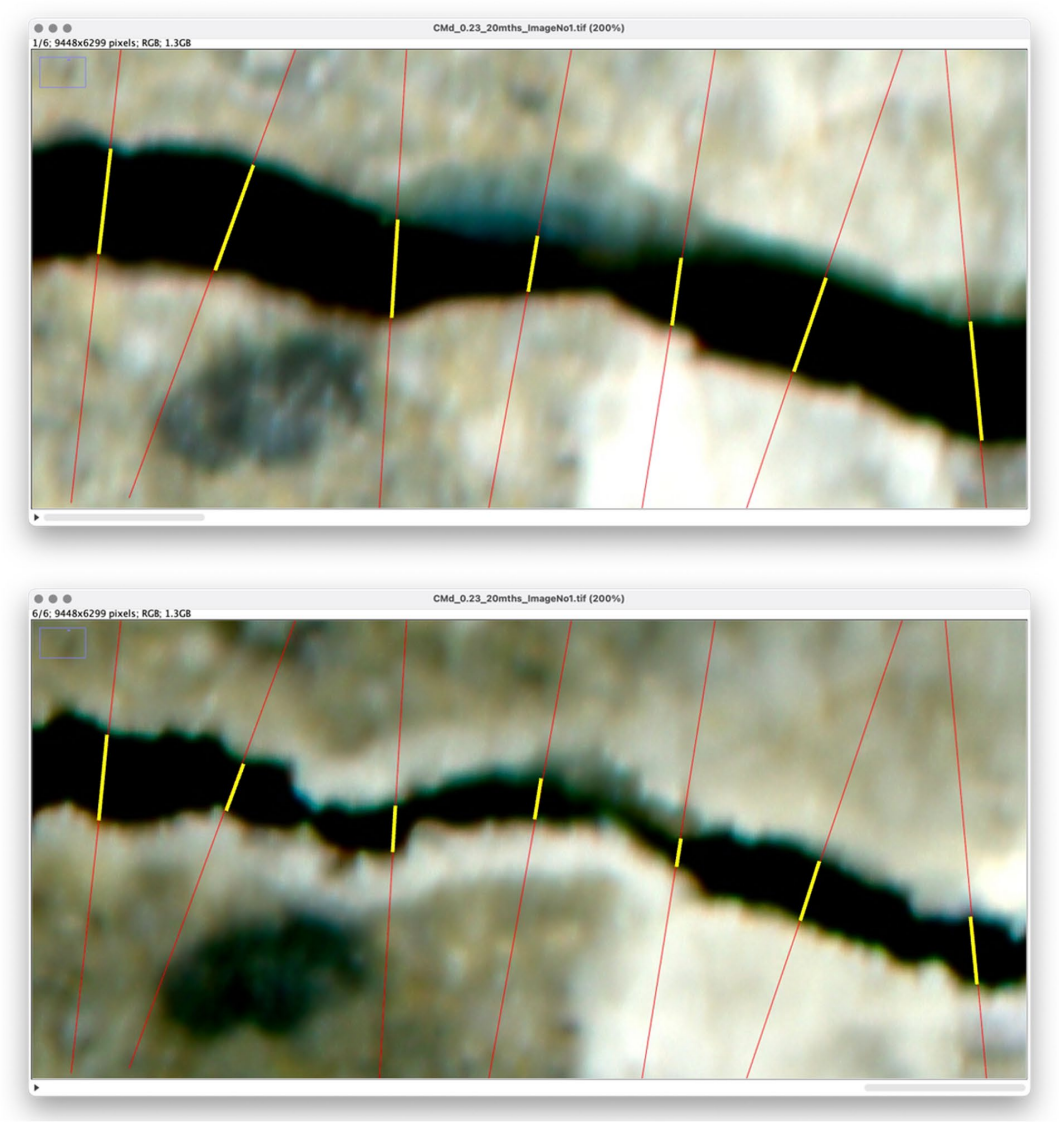
Operator view in ImageJ2 during manual measurements of crack width on concrete surface scans immediately after crack induction (t_1_ = 0, top panel) and after 28 days of self-healing (t_6_ = 28 d, bottom panel); red lines show the grid of intersection lines while yellow lines correspond to the lengths measured by the operator when determining the crack width in their area; in the case shown, the measured crack widths were 127–302 μm for the time point t_1_, and 80–151 μm for the time point t_6_.

In general, manual measurements should be performed in the most natural way possible, under repeatable conditions across the test sample. For the presented dataset, the operator used a 13.3’ Retina matrix monitor with a resolution of 2560 × 1600 pixels, with close to full DCI-P3 gamut coverage and an average fixed screen brightness of 250 nits. During the measurements, the operator was positioned at a distance of approximately 70 cm from the monitor and, due to the glossy coating of the matrix, care was taken to avoid direct light reaching the screen to minimize reflections of light. In ImageJ2, the default zoom level was 200%. For the dataset in this study, the operator aimed to search for points corresponding to the greatest gradient in brightness intensity along the intersection line on each side of the central part of a crack. These points represent transitions in the pixel intensity values that can be interpreted as the boundary between the deeper regions of a crack, typically characterized by darker tones, and the brighter surface of the scanned material sample. If bevelling of the crack edges was observed in the intersection line area, the area interpreted as bevelling (Fig. 2, orange line) would not be included in the crack width measurement. The distance between the detected points which meet the above criterion is then assessed with ImageJ2 GPU. Technically, the process involves connecting the detected characteristic points, which reflect the edges of the cracks, and reading off the length of the line drawn by the operator. If more than one crack is found in the grid-line area, only the width of the crack located in the central part of the grid line is measured. The measurements should be verified and corrected until their repeatability reaches acceptable levels. An example of manual measurements of crack width at different test points is shown in Fig. 4.

### Discussion

The quantitative monitoring of self-healing is challenging regardless of the technique used. Visual crack width measurement is the most common method for quantitative self-healing monitoring. Process monitoring requires the repeated high-resolution measurement of the same specimen in subsequent stages of self-healing, ideally at the same locations. Sufficiently precise assessments are required to detect changes in stage-by-stage crack widths. The measurement conditions, lighting, and surface humidity may affect the crack width assessment results. The self-healing material that fills a crack may appear at different crack depths, and its colour and brightness may change. Moreover, the edges of older cracks are less pronounced and rounder than the edges of fresh scratches and cracks. They are often irregularly and randomly chamfered, making their images and widths fuzzy. The artefacts appearing in the transformed digital images can influence the width estimation results. The filling of a crack through self-healing reduces the crack width and could cause it to disappear. Such fully sealed cracks are difficult to detect with automatic methods and thus influence subsequent statistical analyses when manually identified. Self-healing occurs in heterogeneous and random patterns. Notably, along the same crack, the crack width may decrease in some places and increase in others. Small crack widths are common and important for monitoring self-healing, although accurately measuring them is difficult.

## Data Records

The dataset and images have been shared as a Zenodo record titled “*Dataset for developing deep learning models to assess crack width and self-healing progress in concrete (krkCMd)*”^33^.

The data record include:

1. krkCMd_table.csv: delimited, comma-separated text file containing a resultant dataset of 19,098 crack brightness degree profiles, reference crack width measurements by operator, benchmark crack width measurements by the DLM metasensor and AED detector.
2. krkCMd_images.zip: archive containing source image files in folders by test series:

- stacked images of cracks in subsequent stages of self-healing (.tif files)
- zip archives assigned to image stacks and containing sets of ImageJ data files.roi,
- ImageJ.roi files specifying the locations of grid lines in the images.

3. krkCMd_scripts.zip: archive containing custom scripts supporting image preprocessing and computing benchmark variables.

The total volume of the datafiles is approximately 36 GB.

**Fig. 5:**
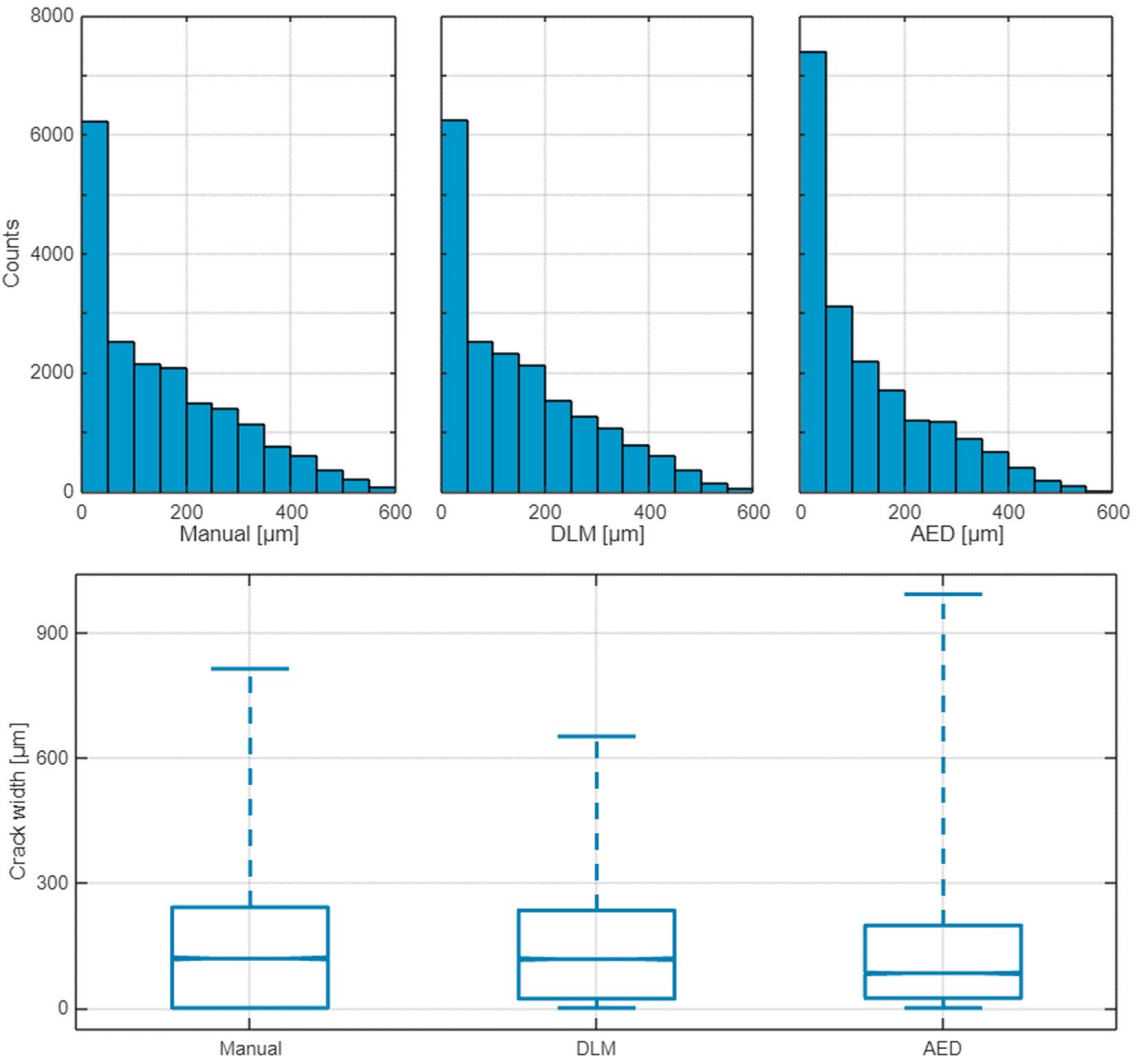
Histograms (top) and boxplots (bottom) of the three crack width measurements related to the 19,098 brightness profiles: measurements by an operator, a DLM, and an AED. The boxplots indicate medians, quartiles, and min and max values.

**Fig. 6:**
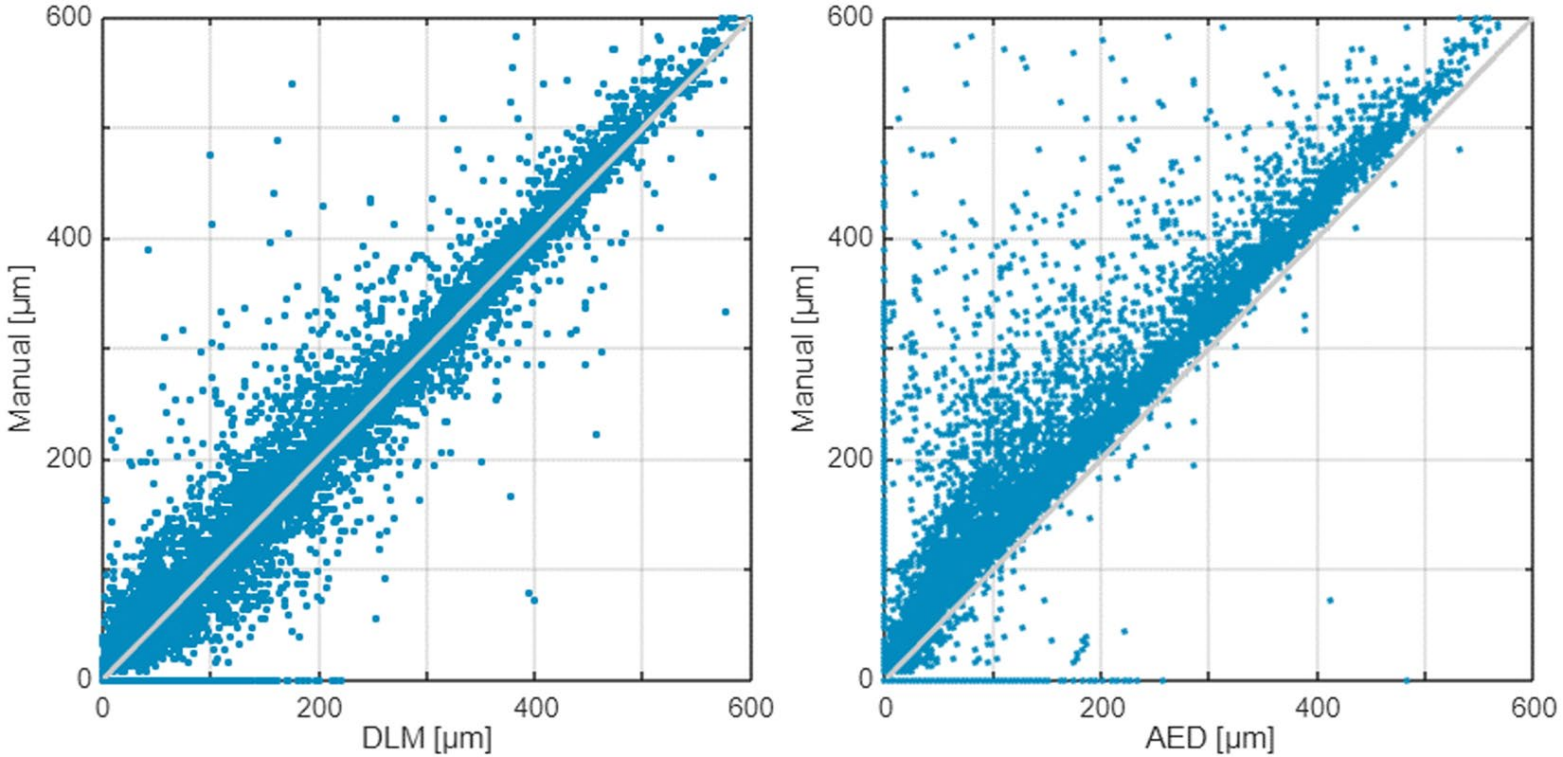
Crack width measurements by a DLM (left) and an AED (right) against manual measurements at 19,098 grid lines.

The dataset consists of 510 variables and 19,098 cases, as shown in Table 3. There are no missing data in this set. Variables 7–507 include the brightness degree profiles extracted from the images along the network of grid lines during the six self-healing stages. Variable 508 (MANwidth) includes manual crack width measurements for the same images and brightness profiles. Manual measurements are obtained from source images along the grid lines and serve as the reference values for developing new deep CNN regression models. Additionally, variables 509-510 (DLMwidth, AEDwidth) include benchmark measurements acquired from the brightness degree profiles obtained using the earlier built DLM and AED models. The histograms, boxplots and scatterplots in Figs. 5–6 characterise the crack width assessment variables 508–510. Source images and grid line data are also provided (.tif,.zip,.roi).

**Table 3:** Structure of the dataset.

Var. No	Var. name	Short description	Type
1	No	Case number; brightness profile number	Integer
2	Profile	Brightness profile unique label, comprising a series, image, grid line and stage labels	String
3	Series	Test series label comprising concrete mix label and self-healing duration	String
4	Image	Image number within test series	Integer
5	Grid	Grid line label	String
6	Stage	Self-healing stage number within test series	Integer
7 … 507	x1 … 501	501 variables representing a 501-pixel-long digital brightness level profile, denoting the brightness degree of the successive pixels along the grid lines transversely crossing the crack mouth. Values range from 0 (black) to 255 (white). Results are interpolated by image preprocessing and stored as real numbers.	Single
508	MANwidth	Manual, reference assessment of crack width by an operator [µm]	Single
509	DLMwidth	Crack width assessment by a deep CNN model^30^ [µm]	Single
510	AEDwidth	Crack width assessment by a threshold-based analytic edge detector [µm]	Single

## Technical Validation

### Factors affecting the extracted brightness profiles and crack width measurements

In the initial stage of developing the methodology for crack width assessment, we investigate the effect of experimental conditions on crack measurements and their uncertainty. To improve the measurement procedure and make it more robust, we examine factors that could affect the measurements. For this research, a composite porous material with a given water absorption capacity is used; notably, differences in the moisture level of the specimen manifest as different changes in brightness levels in different areas. The variability in the moisture content, as well as the colouration of the scanned surface itself, is amplified owing to the presence of carbonate coarse aggregates with different water absorption and colours compared to those of the cement matrix. This creates conditions for greater variability in the surface brightness profiles than is the case when the exterior surface of concrete is covered with a uniform-colour cement slurry. This difference is important for determining crack widths and training CNNs. Moreover, self-healing processes result in the accumulation of material in the volume of cracks and their vicinity, usually with a lighter colour than the surroundings (Fig. 3). Furthermore, positioning a specimen at the same location on the scanner is difficult when scanning the surface at different points in time, as differences could affect illumination angles and images of the crack mouth. Manual measurements are influenced by variations in operator practices and measurement procedure details. In summary, the major random factors that possibly affect the measurements are (1) the specimen position on the scanner, which influences crack illumination, (2) the surface moisture content of the material, which influences brightness, and (3) the operator performing manual measurements.

### Experiments and metrics used

To evaluate the effects of these three factors on the measurements, we perform three experiments, changing one factor at a time. Crack widths are measured through manual, DLM, and AED methods. Each experiment is performed using different specimens and grid-line sets. The data acquisition process and conditions (methodology, image acquisition and preprocessing, type of specimen material, scanner equipment and software used, and measurement instructions) are the same as or similar to those described above. Therefore, this work provides technical validation of the data acquisition methodology and, consequently, the dataset.

Each experiment is performed to assess the effect of different factor on the crack width measurements and measurement uncertainty. Crack widths are measured along the set of grid lines (subjects) at different factor levels; therefore, the measurements are considered multiple dependent samples. The first-choice schema used for comparison is the repeated-measures analysis of variance (ANOVA); however, the corresponding assumptions and the large number of observations and factor levels in this study may justify the use of a nonparametric or multivariate approach. For the three experiments, the repeated-measures ANOVA F-test, as well as the Friedman test and Wilks test, indicate statistically significant differences among the facto level groups with p-values less than 0.00001. Tests based on large dependent sample sets even slight differences classify as statistically significant, therefore, we focus on effect size. To quantify effect size, generalized eta-squared^34^ (${\eta }_{G}^{2}$) is used, which is a proportion of variance based index. Its values are ranging from 0 to 1 and reflect an increasing discrimination of measurements and increasing effect of the factor on measurements. Generalized eta-squared is appropriate for dependent samples analysis, allows for comparisons across different designs and can be interpreted against the Cohen’s benchmarks^35,36^, therefore, its use is justified for this study. Boxplots illustrate the measurement discrimination by factor level. Scatterplots illustrate the dispersion around the subject means and the measurement variability not explained by the subjects. The root-mean-square deviation (*RMS*), which is an absolute measure of this variability, is used to estimate standard measurement uncertainty and reproducibility.

1 $$RMS=\sqrt{\frac{1}{NM}\mathop{\sum }\limits_{i}^{N}\mathop{\sum }\limits_{j}^{M}{({w}_{j}^{i}-{\bar{w}}_{j})}^{2}}$$

2 $${\eta }_{G}^{2}=\frac{N{\sum }_{j}^{M}{({\bar{w}}_{j}-\overline{\overline{w}})}^{2}}{{\sum }_{i}^{N}{\sum }_{j}^{M}{({w}_{j}^{i}-\overline{\overline{w}})}^{2}}$$

where *N* is the number of factor levels (groups); *M* is the number of grid lines (subjects); ${w}_{j}^{i}$ is the crack width measurement for the *i*-th factor level along the *j*-th grid line; ${\bar{w}}_{j}$ is the mean crack width along the *j*-th grid line; ${\bar{w}}^{i}$ is the mean crack width for the *i*-th factor level sample; and $\overline{\overline{w}}$ is the grand mean. The formulas above apply for dependent samples (repeated measures) designs.

Authors interpret *RMS* value below 20 µm as low and above as moderate. Effect sizes ${\eta }_{G}^{2}$ are interpreted using benchmarks provided by Cohen^35^: small value 0.01, medium value 0.06, large value 0.14. These benchmarks we consider low^36,37^, however, they allow to distinguish qualitative descriptions of the ${\eta }_{G}^{2}$ values, small in this experiment.

### Effects of the factors on measurements

**Fig. 7:**
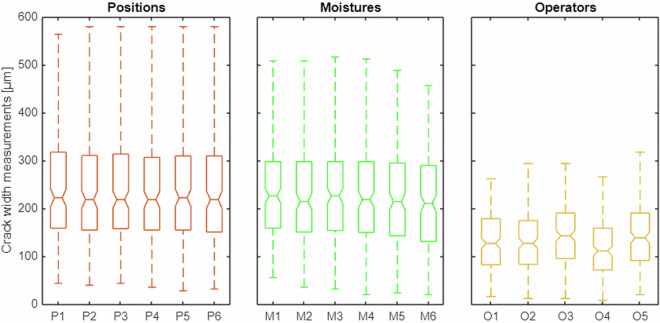
Boxplots illustrating the group statistics for the three experiments (position, moisture, and operator). The medians, quartiles, minimum and maximum values of the manual measurements by factor level are displayed.

**Fig. 8:**
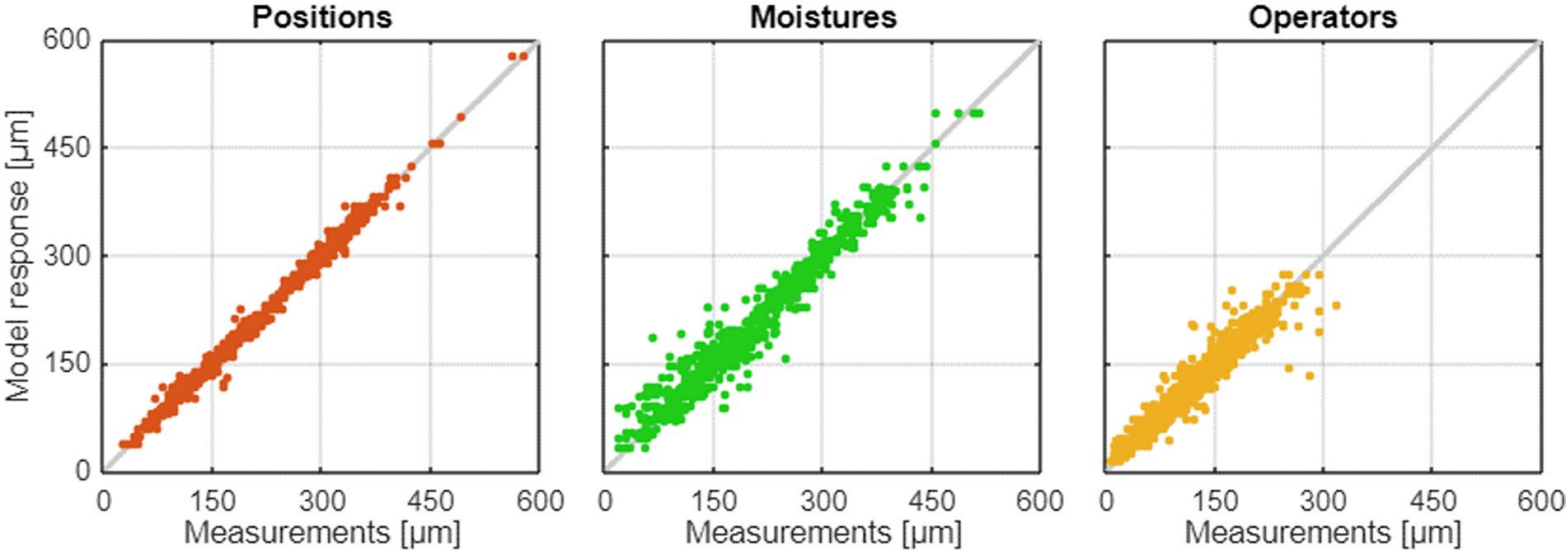
Scatterplots by factor (position, moisture, and operator). Each point indicates the manual crack width measurement vs. the mean measurement along the same grid line.

Three experiments are performed on the CMd_0.28 concrete specimens (Table 1). The first experiment is performed to assess the effect of inaccuracies in the position of the specimen on the scanner plate, resulting in different crack illumination levels. The specimen is scanned six times, with its orientation changed within an angle range of 10° and the location changed by 4 cm or less. Subsequently, operator performs manual crack width measurements along the grid lines (subjects) at all positions (factor levels). Boxplots by position show the homogeneity of the plotted group statistics (Fig. 7). However, the boxplot statistics do not consider group dependency, unlike the repeated-measures ANOVA. A generalized eta-squared of 0.0003 (Table 4) indicates small effect of specimen position on the manual measurements. An *RMS* of 7.4 µm and a tightly linearly arranged scatterplot (Fig. 8) indicate low measurement uncertainty. In addition, the DLM and AED measurements demonstrate a small effect size and low measurement uncertainty (Table 5).

**Table 4:** Effects of the experimental factors (specimen position on the scanner, moisture level, and operator) on manual measurements. Factor effect size (${\eta }_{G}^{2}$) and dispersion statistic (*RMS*).

1	Crack width measurement method	Manual		
2	Factor	Position	Moisture	Operator
3	Sample size/number of factor levels	1134/6	1134/6	945/5
4	Generalized eta-squared ${\eta }_{G}^{2}$	0.0003	0.0049	0.0292
5	Root mean square deviation *RMS* [µm]	7.4	18.2	16.6

**Table 5:** Effects of specimen position and moisture level on DLM and AED measurements.

1	Crack width measurement method	DLM		AED	
2	Factor	Position	Moisture	Position	Moisture
3	Sample size/number of factor levels	1134/6	1134/6	1134/6	1134/6
4	Generalized eta-squared ${\eta }_{G}^{2}$	0.0004	0.0163	0.0008	0.0236
5	Root mean square deviation *RMS* [µm]	7.8	25.5	11.3	29.4

Factor effect size (${\eta }_{G}^{2}$) and dispersion statistic (*RMS*).

The second experiment is performed to evaluate the effect of specimen saturation and brightness on the measurements. Based on the results, the effect size is small (0.0049). Boxplots show the similarities in group statistics. An *RMS* of 18.2 µm and the scatterplot indicate low measurement dispersion. Furthermore, the DLM and AED measurements show small to medium effect size and moderate measurement uncertainty.

The third experiment is used to examine the measurements by the five operators for the same set of grid lines. The effect size is 0.0292 (Table 4), the measurement dispersion *RMS* is 16.6 µm. These results indicate small to medium differences among the operators, low uncertainty and high reproducibility of the measurements. In this experiment, all operators measure the crack widths from the same scans and grid lines; therefore, the DLM and AED measurements do not depend on the operator and the operator effect analysis is not applicable.

The three experiments are used to examine the measurement methodology applied to produce the dataset. The methodology, spanning several stages and interim procedures, is evaluated based on the resultant measurements. The results indicate that inaccuracies in positioning the specimen on the scanner and the related changes in illumination have a small effect on the measurements. The specimens should be carefully positioned on the scanner at each subsequent stage of self-healing, however, the procedure in use is sufficient.

The measurement method is based on the visual assessment of crack widths. Therefore, diverse specimen surface moisture levels might affect the homogeneity and dispersion of measurements. The results show that the effect size and uncertainty are small or small to medium. However, a more rigorous procedure for drying and ensuring the same surface moisture level has been used for collecting the dataset presented in the data descriptor.

Although operators clearly influence crack width measurements, the study results show that the effect size and measurement uncertainty are small to medium. The results show that to obtain consistent and reproducible results, all measurements do not need to be performed by the same operator. However, the measurements in the dataset are obtained by the same trained operator following the same measurement procedure. In summary, the results of the three experiments show that the measurement uncertainty and sensitivity considering difference factors is small to medium. Thus, the experiments positively validate the measurement methodology that assures the quality of the dataset.

## Usage Notes

### Using the data for training and testing deep CNNs

The presented dataset is suitable for developing deep CNN models for measuring crack widths based on crack brightness degree profiles. Brightness profiles and manual reference measurements are included. The stacked source images coupled with grid line files are provided, allowing users to repeat the procedures performed by the authors or to carry out multitude of other operations. This also enables users to avoid time-consuming laboratory work and manual reference measurements and to focus on self-healing data analytics or deep CNN model building, hyperparameter optimisation, and model deployment. The same set of crack widths is measured based on the provided brightness profiles using the authors’ own CNN metasensors and analytic threshold-based crack edge detectors. The results are provided in the dataset as supplementary variables for benchmarking. Since the dataset is the result of an experimental study of concrete self-healing, it can also be used for self-healing studies.

### Limitations

Based on the characteristics of the source images resulting from the use of constant lighting conditions and a single resolution when imaging the surface of the specimens, the results of this study may not align with those of analogous tests based on images using other types of equipment, such as digital cameras or smartphones. Furthermore, the results are based on the testing of high-strength concrete containing light- and dark-coloured coarse aggregates. This study did not test composites with significantly different compositions, such as those containing only microaggregates or reinforcing fibres. Therefore, the results may be affected by factors not considered in the presented study.

## Data Availability

Image preprocessing and crack width measurements are performed using the ImageJ2 (version 2.9.0/1.53t) programming environment^31^. The image registration process is carried out using the SIFT procedure^32^ implemented in ImageJ. The set of used registry parameters is included in Table 2. The Zenodo record^33^ contains scripts supporting image preprocessing and computing benchmark variables.
